# Candidal colonization, strain diversity, and antifungal susceptibility among adult diabetic patients

**DOI:** 10.4103/0256-4947.60514

**Published:** 2010

**Authors:** Safia A. Al-Attas, Soliman O. Amro

**Affiliations:** From the Department of Oral Basic and Clinical Sciences, King Abdul Aziz University Faculty of Dentistry, Jeddah, Saudi Arabia

## Abstract

**BACKGROUND AND OBJECTIVES:**

Candidal colonization in diabetics is a matter of debate. The aim of this study is to investigate oral candidal colonization, strain diversity, antifungal susceptibility, and the influence of local and systemic host factors on candidal colonization in adult diabetics.

**METHODS:**

We conducted a case-control study that compared 150 diabetics (49 type 1, 101 type 2) with 50 healthy controls. Two salivary samples were collected, using the oral rinse sampling method: one for salivary flow rate and pH determination, and the other for candidal colonization assessment. The candidal isolates were identified and tested in vitro for antifungal susceptibility using the commercial kit, Candifast. The relationship between specific host factors and candidal colonization was also investigated.

**RESULTS:**

Diabetics had a higher candidal carriage rate compared to controls, but not density. *Candida albicans* was the most frequently isolated species, but diabetics had a variety of other candidal species present. None of the control samples were resistant to any tested antifungal, while the diabetic samples had differing resistances to azole antifungals. Although there was a significant positive correlation between glycemic control and candidal colonization in type 2 diabetics, there was a negative correlation between salivary pH and candidal carriage in the controls versus density in type 2 diabetics.

**CONCLUSION:**

Diabetic patients not only had a higher candidal carriage rate, but also a variety of candidal species that were resistant to azole antifungals. Oral candidal colonization was significantly associated with glycemic control, type of diabetes, and salivary pH.

Oral candidiasis is a common opportunistic infection of the oral cavity caused by an overgrowth of candidal species, the most common being *Candida albicans*.^1^ The underlying causes of oral candidiasis include extremes of age, xerostomia, antibiotic therapy, dentures, smoking, Cushing syndrome, malignancies, immune deficiencies, and diabetes mellitus (DM).^1^,^2^ The presence of *Candida* species and the density of candidal growth in the oral cavity is often said to be increased in patients with DM.^3^,^4^ However, these observations remain controversial.^5^ Similarly, there are conflicting reports on the identity of the underlying risk factors.^3^–^5^ These uncertainties are thought to be related to the different pathophysiological behaviors of the two clinical types of DM, to different patients and disease data such as, age, duration or control of diabetes or, at least in part, are thought to reflect racial and environmental differences among diabetic populations worldwide.

Diabetes mellitus, specifically type 2 diabetes, is a major public health issue. The diabetic population is expected to increase from 171 million in 2000 to 366 million by 2030.^6^ World Health Organization statistics show that, worldwide, almost three million deaths per year are attributed to diabetes, equivalent to 5.2% of all deaths.^7^ DM is emerging as a major public health problem in Saudi Arabia, parallel with the worldwide diabetes pandemic. Early reports indicated that nearly one Saudi in five of age 30 years or older had DM,^8^ while the latest report showed that the overall prevalence of DM among adult Saudis of both sexes in rural as well as urban communities is 23.7%.^9^ Despite these recognized high rates of DM among the Saudi population, there is an obvious lack of published studies evaluating the prevalence and characteristics of oral fungal infections, or investigating the underlying risk factors associated with DM, in Saudi Arabia. The aim of this study is to investigate oral yeast colonization (rate and density of acquisition of *Candida* species colonies), strain diversity, and antifungal susceptibility in adult diabetics, and to evaluate the influence of some local and systemic host factors on candidal colonization.

## METHODS

This case-control study was conducted on 150 diabetic patients (49 type 1, 101 type 2) and 50 healthy controls. The diabetic patients were recruited from the diabetic clinics of King Abdul-Aziz University Hospital (KAAUH), Jeddah, Saudi Arabia, during routine diabetic follow-up appointments. Consecutive eligible patients identified on a specific sampling day were asked to participate. The inclusion criteria were a diagnosis of either type 1 or 2 DM and age of 18 years or older. The control group included age-grouped and sex-matched healthy volunteers (companions of the diabetic patients as well as dental auxiliaries) with no history of diabetes, who were selected from KAAUH. All subjects in both groups gave a signed informed consent to participate in the study. Individuals who had received antibiotics or steroid therapy or had been using antiseptic mouthwash during the prior three weeks were excluded from the study. The study protocol was approved by the ethics committee of the KAAUH medical faculty.

A structured questionnaire was developed for collecting information on demographics (age and gender), medical variables (diabetes type, duration, and presence of diabetes-related systemic diseases), and local factors (denture status, oral hygiene, and smoking). The investigators supervised the completion of the questionnaire, which had close-ended questions including options. The patient's medical records were used to gather information on diabetes type and duration, as well as, the presence of diabetes-related systemic complications (retinopathy, nephropathy, neuropathy, and peripheral vascular diseases). The presence of any of these complications was considered a positive finding and dichotomized on a scale of yes or no. All subjects provided three samples, two salivary and one blood sample, which were sent immediately to the KAAUH Microbiology and Hematology Laboratories, respectively. To prevent circadian variations, the samples were collected between 9 a.m. and 12 noon.

The subject was typically instructed not to eat, drink or smoke two hours prior to sample collection. They were asked to lean their upper body forward, and allow oral fluid to drip into a graduated collection vial, over a five-minute period, without swallowing.^10^ This sample was used to calculate the salivary flow rate (mL/min) and to determine the saliva pH using the Combur test (Roche Diagnostics Ltd., UK), according to the manufacturer's instructions. Oral yeast colonization was assessed microbiologically in all participants, regardless of the presence of clinical infection. An oral rinse technique described by Samaranayake and colleagues 11 was used for sample collections. Each subject was supplied with 10 mL sterile phosphate-buffered saline (0.1 M PBS, pH 7.2) in a universal container, requested to remove dentures if worn, and to swirl the 10 mL of PBS around the mouth for 60 seconds before expectorating the saliva-buffer mixture back into the container. The sample was sent immediately to the microbiology laboratory and inoculated onto a Sabouraud's dextrose agar (SDA) plate, and incubated aerobically at 37°C for 48 hours. The growth of any candidal colonies was recorded as a positive growth and the subject as a candidal carrier. The number of colonies on each plate was counted and the number of colony-forming units (CFU) per mL calculated, to indicate candidal density. The *Candida* strains in the isolated colonies were identified and tested for susceptibility to amphotericin B, fluconazole, nystatin, flucytosine, econazole, ketoconazole, and miconazole antifungals, using the commercial Candifast kit (International Micobio, France) according to the manufacturer's instructions. Basically, the Candifast test tray consisted of two rows with eight wells. The first row (the identification row) contained seven different sugars, the fermentation of which produced a color change in the phenol red indicator. The first well of this row contained phenol red, actidione, and glucose. Interpretation help was given by a colored chart included in the kit. The second row was the susceptibility row. The first well of this row was a growth control well and contained glucose. Wells two to eight contained glucose and each with contained an antifungal agent. The wells were inoculated with standardized inocula and covered with two drops of paraffin oil. The tray was incubated at 37°C. After 24 and 48 hours, the wells containing antifungal drugs (row 2) were examined, and the isolate was classified as ‘resistant’ (medium color: yellow and/or visible turbidity and/or a pellet), ‘intermediate’ (medium color: yellow-orange) or ‘susceptible’ (medium color: red). The minimal inhibitory concentrations (MICs) corresponding to these three categories were not provided. Additionally, the identification of *Candida* species was confirmed by the conventional methods of germ tube production in horse serum,[12] following incubation at 35°C for two hours.

For diabetic patients, the blood sample was used to measure glycosylated hemoglobin concentrations (HbA_1c_), which assessed the long-term glycemic control of that patient. For control subjects, the blood samples were used to measure the fasting plasma glucose level. Those with values of 7 mmol/mL or higher were excluded from the study, according to the WHO definition of diabetes.^13^

## RESULTS

**Figure 1:**
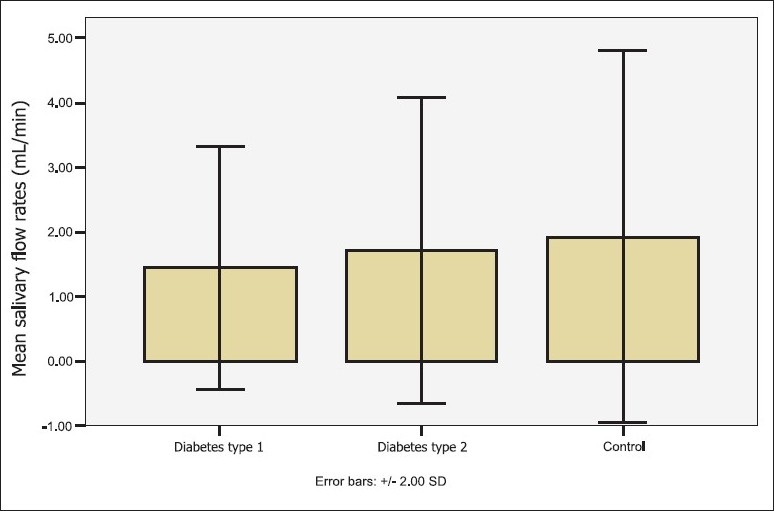
Salivary flow rates among the groups.

The diabetic and control groups were homogenous in terms of age, sex, dental status, and smoking habits (*P>*.05; Table 1). However, the control group showed a higher tooth brushing frequency than the diabetic group (*P=*.001). Also, there were no statistically significant differences between the two types of diabetic patients in any of the above-mentioned variables, nor in disease history, salivary flow rate or pH, except for the age, brushing frequency, and glycemic control (Tables 1 and 2). Type 2 diabetics were older than type 1 diabetics and had both a lower brushing frequency and glycosylated hemoglobin levels in comparison to type 1 diabetics [mean (SD) HbA_1c_ =8.95 (1.78) compared to 10.06 (2.06), respectively]. The diabetics had statistically, significantly lower salivary pH values compared to the controls (*P*=.015). However, there were statistically no significant differences in the levels of the salivary flow rates among the groups (Table 2, Figure 1).

**Table 1:** Characteristics of the study population.

	Diabetics (n=150)	Controls (n=49)	P*	Diabetics		P*
				Type 1 (n=49)	Type 2 (n=101)	
Age (years)						
<30	12.7	14.0		32.7	3.0	
30-50	37.3	52.0	.128	30.6	40.6	.001
>50	50.0	34.0		36.7	56.4	

Sex						
Male	41.3	38.0	.678	34.7	44.6	.250
Female	58.7	62.0		65.3	55.4	

Brushing frequency						
<1/day	16.0	4.1		12.8	17.5		
Once/day	37.5	18.4	.001	36.2	38.1	.673
>2/day	46.5	77.6		51.1	44.3	

Dental status						
Dentate	80.0	86.0	.346	84.8	77.8	.326
Denture wearer	20.0	14.0		15.2	22.2	

Smoker						
Yes	10.1	10.0	.98	12.8	8.9	.47
No	89.1	90.0		87.2	91.1	

Values are percentages;

* Chi-square test

**Table 2:** Candidal carriage, density, salivary flow rates, and pH among the groups.

	Diabetics	Controls	*P*	Diabetic patients		*P*
				Type 1	Type 2	
Candidal carriage						
Positive %	33.3	14.3	.028	51.2	25	.003
Negative %	66.7	85.7		48.8	75	
Candidal density (CFU/mL)						
Median (IQR)	0.0 (2000)	0.0 (0.0)	.200	1000 (3000)	0.0 (750)	.002
Salivary flow rates (mL/ mints)						
Median (IQR)	1.5 (1)	1.5 (1.13)	.479	1.0 (1.3)	1.5 (1)	.195
pH						
Median (IQR)	7 (2)	7 (1.0)	.015	7 (1)	7 (2)	.059

IQR: Interquartile range

**Figure 2:**
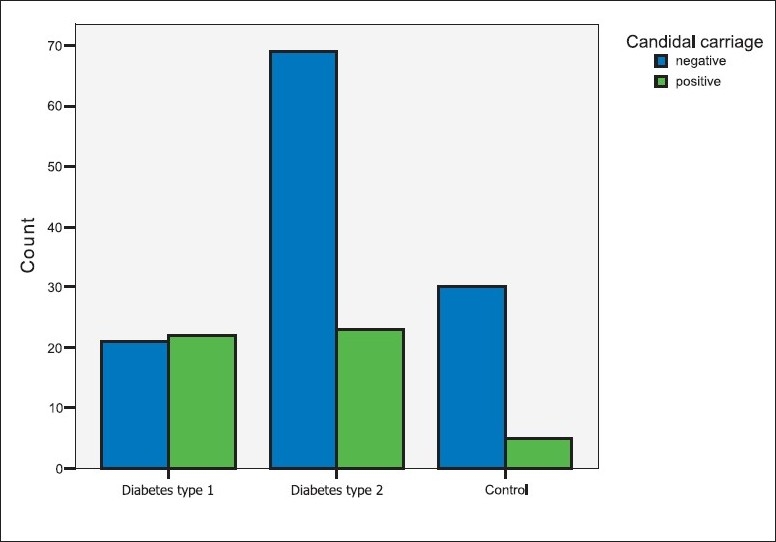
Candidal carriage among the groups.

**Figure 3:**
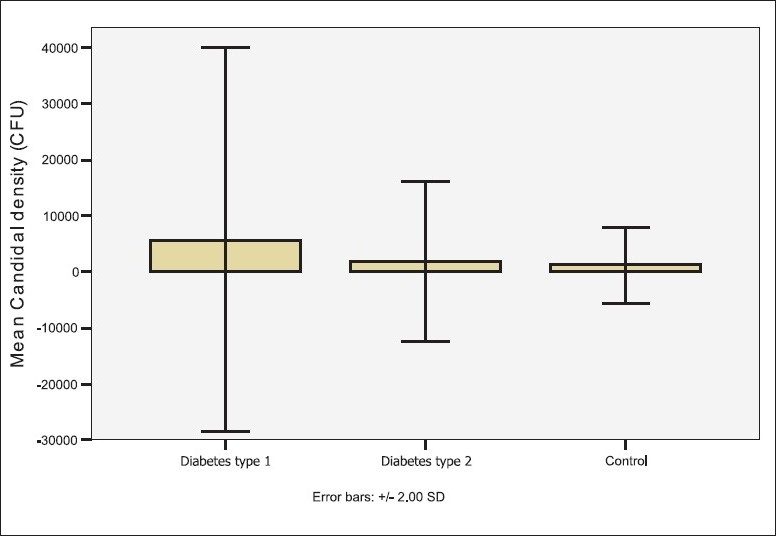
Candidal density among the groups.

The carriage rate or the frequency of detecting positive *Candida* growth was significantly higher in the diabetic patients than in the controls (*P=*.028; Table 2, Figure 2). The diabetic patients who were candidal carriers harbored the yeast in higher densities than the control carriers, but the difference was not statistically significant (Figure 3). The mean candidal density for diabetic patients in this study was 3140.74 CFU/mL (SD=11388.76). The number of patients with candidal carriage from the oral cavity was higher in patients with type 1 diabetes than in type 2 (*P=*.003). Similarly, the yeast density was higher in type 1 diabetics than in samples from type 2 diabetics, and the differences between the two groups were statistically significant (*P=*.002).

In both diabetic patients and healthy controls, the most frequently isolated yeast was *Candida albicans* (68.9% and 40%, respectively). The remaining isolates were found more frequently in DM patients as compared to the controls, including *C glabrata* (11.1%), *C parapsilosis* (6.7%), *C krusei* (4.4%), *C tropicalis* (2.2%), and other yeast species (6.7%) (Table 3). The in vitro antifungal susceptibility testing revealed that the yeast isolated from the diabetic patients had different rates of resistance to the seven tested antifungal drugs, except amophotericin B and nystatin, against which they had no resistance (Table 4). In contrast, in the healthy controls, none of the isolated yeast showed any resistance to the tested antifungal agents. When patients with different types of DM were compared, there was statistically no significant difference in the antifungal susceptibility (*P*>.05).

**Table 3:** Distribution of candidal isolates in diabetics and healthy controls.

Candidal spp.	Diabetics	Controls	Diabetic patients	
			Type 1	Type 2
*C albicans*	31 (68.9)	2 (40.0)	14 (63.6)	17 (73.9)
*C parapsilosis*	3 (6.7)	0 (0.0)	1 (4.5)	2 (8.7)
*C glabrata*	5 (11.1)	1 (20.0)	3 (13.6)	2 (8.7)
*C krusei*	2 (4.4)	0 (0.0)	2 (9.1)	0 (0)
*C tropicalis*	1 (2.2)	0 (0.0)	1 (4.5)	0 (0)
Other yeast spp	3 (6.7)	2 (40.0)	1 (4.5)	2 (8.7)

Values are numbers and percentages

**Table 4:** In vitro susceptibility to antifungal agents.

Antifungal	Diabetics	Controls	Diabetic patients	
			Type 1	Type 2
Amphotericin B				
S	37 (100)	2 (100)	21 (100)	16 (100)
R	0 (0)	0 (0)	0 (0)	0 (0)

Nystatin				
S	37 (100)	2 (100)	21 (100)	16 (100)
R	0 (0)	0 (0)	0 (0)	0 (0)

Flucytosine				
S	31 (86.1)	2 (100)	17 (81)	14 (93.3)
R	5 (13.9)	0 (0)	4 (19)	1 (6.7)
Fluconazole				
S	25 (71.4)	2 (100)	14 (73.7)	11 (68.8)
R	10 (28.6)	0 (0)	5 (26.3)	5 (31.3)
Ketoconazole				
S	30 (85.7)	2 (100)	17 (85.)	13 (86.7)
R	5 (14.3)	0 (0)	3 (15)	2 (13.3)

Miconazole				
S	24 (68.6)	2 (100)	14 (73.7)	10 (62.5)
R	11 (31.4)	0 (0)	5 (26.3)	6 (37.5)

Econazole				
S	19 (73.1)	1 (100)	11 (73.3)	8 (72.7)
R	7 (26.9)	0 (0)	4 (26.3)	3 (27.3)

Values are numbers and percentages; S: sensitive, R: resistance

**Table 5:** Factors potentially influencing candidal growth in type 2 diabetics.

	Positive candidal growth		Negative candidal growth	
	Type 2 diabetics	Controls	Type 2 diabetics	Controls
HbA_1c_	9.90 (1.88)*P=.006	NA	8.72 (1.65)*P=.006	NA
Salivary pH	6.8 (0.91)*P=.139	6.40 (0.55)*P=.003	7.10 (0.82)*P=.139	7.37 (0.63)*P=.003

Data are mean (SD); NA= not applicable

* ANOVA

Statistical analysis showed no significant relationship between either the rate or density of candidal carriage and age, sex, brushing frequency, denture status, or smoking habits in any of the studied groups (*P*>.05). Similarly, medical and dental parameters such as disease duration, presence of diabetes complications, and salivary flow rates did not correlate significantly with the *Candida* carriage rate or density (*P*>.05). However, the rate of candidal carriage correlated significantly with the degree of glycemic control (as determined by the HbA_1c_ values) in type 2 diabetics and the salivary pH levels in healthy controls (Table 5). The candidal carriage rate correlated positively with the Hb_A1c_ values in type 2 diabetics, but negatively with the pH values in the healthy controls. In the type 2 diabetics, the candidal density correlated positively with the HbA_1c_ values (Pearson correlation=0.212; *P=*.047), and negatively with the salivary pH values (Pearson correlation=−0.338; *P=*.001).

## DISCUSSION

A review of the literature published to date, on the relationship between DM and oral candidal infection reveals a continuing debate. Studies on candidal colonization are often contradictory, which may be a result of the variety of sampling techniques employed.^14^–^18^ The present study comprehensively investigated microbiologically oral yeast colonization and evaluated the effect of some local and systemic factors, which could potentially influence the candidal carriage rate and density in diabetic patients. The reason for not conducting an oral examination for the clinical diagnosis of *Candida* was that it could be counted microbiologically without manifesting clinically and this was mentioned in the literature. A significant proportion of patients with no clinical evidence of oral candidiasis had >100 CFU/mL of yeast in their mouth rinses, suggesting that even an abundance of organisms in saliva might not initiate candidal infection.^5^ The oral rinse sampling method was used as it was the most appropriate and sensitive technique for evaluating the overall yeast carriage compared to imprint culture, swab or saliva sampling.^11^ The percentage of diabetic candidal carriers was nearly double that of healthy controls, confirming previous reports that candidal species were more prevalent in the oral cavity of diabetic patients than in healthy individuals.^4^,^14^,^17^ In addition, the prevalence rates for candidal species in the present study, of 33.3% in diabetics compared with only 14.3% in the healthy controls, are similar to those in the previous studies, which reported a range of 18 to 80% for diabetic patients and 3 to 47% for healthy individuals.^5^,^14^ The mean candidal density for the current diabetic isolates was 3140.74 CFU/mL, a value also within the range of candidal density reported among diabetic adult populations.^3^,^19^

Overall, our results herein agree with the other published results using the same sampling techniques. Diabetic patients have a higher prevalence of oral candidal carriage rate, but not density, compared to non-diabetics.^14^,^19^,^20^ Although candidal density seems to be a useful predictor for the development of oral candidiasis in diabetic patients, previous studies have shown that candidal density, regardless of the methods used for sampling, does not correlate with the clinical evidence of oral candidiasis.^21^ The higher carriage rate that we found in our study may be a better predictor for candidal infection in diabetics, but the question needs further investigation. The present study also agrees with the previously published reports that oral yeast colonization is higher in type 1 diabetics than in type 2,^4^,^15^ although some investigators have failed to show a correlation between candidal carriage rates and density or the type of DM and the treatment used.^14^,^18^,^22^

*C albicans* was the most frequently isolated candidal species among the study groups, even as 31.1% of diabetic patients also carry other yeasts and candidal species. The latter include *C glabrata* (11.1%1), *C parapsilosis* (6.7%), *C krusei* (4.4%), and *C tropicalis* (2.2%). These findings are not surprising, as these organisms are the most common candidal species isolated from humans.^23^ A similar diversity in candidal species has been reported previously among diabetic populations in Thailand,^24^ Poland,^25^ and Brazil.^26^

The most common antifungal drugs in the current clinical use, for treatment of oral candidiasis are polyenes (amphotericin B and nystatin) and azoles (miconazole, fluconazole, ketoconazole, and itraconazole), mainly used topically.^27^ The therapeutic and prophylactic use of antifungal agents has given rise to alarming cases of antifungal resistance.^28^ Interestingly, in vitro antifungal susceptibility tests revealed that none of the candidal isolates from the control group showed any resistance to the seven tested antifungals. However, oral yeasts isolated from diabetic patients displayed different resistance rates from the five azole antifungal agents, mainly miconazole and fluconazole. Such findings confirmed the results of other researchers reporting the emergence of fluconazole and, more generally, triazole resistance among different groups of immunocompromized patients in whom these agents were frequently used.^29^ A recent in vitro study using the commercial Fungitest kit revealed no difference in susceptibility between candidal isolates from diabetics and non-diabetics to the six common antifungal agents tested.^27^ However, they reported increased miconazole resistance among diabetic patients in London, which they justified as a consequence of the past widespread use of miconazole for the treatment of *Candidal* induced denture stomatitis. In recent times, a number of rapid and easy-to-use commercial products have been introduced that identify yeast, and test antifungal susceptibility. The Candifast kit used in this study proved to be reliable for the identification of yeasts, but it had low reliability in antifungal susceptibility testing, which was attributed to high levels of subjectivity in the result interpretations.^30^ However, the objective of this study was not to report accurate and clinically significant susceptibility, but to investigate whether there was an increase in in vitro antifungal resistance in the diabetic isolates. Therefore, this study should be extended to include more precise standardized tests with clear-cut objectives and interpretative criteria. Overall, based on the above findings we can say that the accurate identification of strains isolated from diabetic patients was especially important, because they were more likely to carry species other than *C albicans*, which might not be sensitive to certain antifungal agents. In addition the culture and sensitivity testing would add to the value of selecting the appropriate antifungal drugs, rather than prescribing any type of antifungal just for the presence of clinical manifestations of candidal infection.

The risk factors for oral candidal infection in diabetic patients are complex. Some authors emphasize the role of local factors such as low salivary flow rates^19^ and pH,^15^ smoking,^3^,^21^ wearing dentures^15^,^22^,^31^ and poor oral hygiene,^22^ others focus on systemic factors such as patient age^22^ or disease types,^4^ degree of control,^15^,^31^ and the presence of complications.^31^ Overall, our results demonstrate that oral yeast carriage rate and density are not influenced significantly by patient age, gender, disease duration or complications. No statistically significant relationship was found between smoking habits, wearing dentures, brushing frequency, salivary flow rates and carriage rates or the density of candidal isolations. However, glycemic control, as determined by the level of glycosylated hemoglobin, positively correlated with the increased carriage rate and density of *Candida* isolates in type 2 diabetics. This may explain the higher carriage rates reported among type 1 diabetics compared to type 2 diabetics, because the former had higher mean HbA_1c_ values and poorer glycemic control. Hyperglycemia could contribute to the risk of oral *Candida* infection by increasing salivary glucose levels, which may promote overgrowth of *Candida*.^31^ The results reveal that decreased salivary pH correlates with an increased candidal carriage rate in healthy controls, and increased candidal density in type 2 diabetics. Interestingly, although similar findings regarding salivary pH and glycemic control have been reported,^15^ they have failed to show a significant difference in the candidal carriage rates or density between diabetics and non-diabetic controls.

In conclusion, diabetic patients had a higher oral candidal carriage rate, but not density, compared to non-diabetic controls. Although *C albicans* was the predominant isolate, a variety of other candidal species, with less susceptibility to azole antifungals, were identified in diabetics. Oral candidal colonization was significantly associated with diabetic type, glycemic control, and salivary pH, demonstrating a potential role of these factors in controlling candidal infections.

## References

[1] Akpan A, Morgan R (2002). Oral candidiasis. Postgrad Med J.

[2] Rossie K, Guggenheimer J (1997). Oral candidiasis: Clinical manifestations, diagnosis, and treatment. Pract Periodont Aesthet Dent.

[3] Willis AM, Coulter WA, Fulton CR, Hayes JR, Bell PM, Lamey PJ (1999). Oral candidal carriage and infection in insulin-treated diabetic patients. Diabet Med.

[4] Kumar BV, Padshetty NS, Bai KY, Rao MS (2005). Prevalence of *Candida* in the oral cavity of diabetic subjects. J Assoc Physicians India.

[5] Soysa NS, Samaranayake LP, Ellepola AN (2006). Diabetes mellitus as a contributory factor in oral candidosis. Diabet Med.

[6] Wild S, Roglic G, Green A, Sicree R, King H (2004). Global prevalence of diabetes: Estimates for the year 2000 and projections for 2030. Diabetes Care.

[7] Roglic G, Unwin N, Bennett PH, Mathers C, Tuomilehto J, Nag S (2005). The burden of mortality attributable to diabetes realistic estimates for the year 2000. Diabetes Care.

[8] El-Hazmi MA, Warsy AS, Al-Swailem AR, Al-Swailem AM, Sulaimani R, Al-Meshari AA (1996). Diabetes mellitus and impaired glucose tolerance in Saudi Arabia. Ann Saudi Med.

[9] Al-Nozha MM, Al-Maatouq MA, Al-Mazrou YY, Al-Harthi SS, Arafah MR, Khalil MZ (2004). Diabetes mellitus in Saudi Arabia. Saudi Med J.

[10] Navazesh M (1993). Methods for collecting saliva. Ann N Y Acad Sci.

[11] Samaranayake LP, MacFarlane TW, Lamey PJ, Ferguson MM (1986). A comparison of oral rinse and imprint sampling techniques for the detection of yeast, coliform and *Staphylococcus aureus* carriage in the oral cavity. J Oral Pathol.

[12] Mackenzie DW (1962). Serum tube identification of *Candida albicans*. J Clin Pathol.

[13] World Health Organization (2006). Definition and Diagnosis of Diabetes Mellitus and Intermediate Hyperglycimia: Report of a WHO/IDF Consultation.

[14] Lamey PJ, Darwaza A, Fisher BM, Samaranayake LP, Macfarlane TW, Frier BM (1988). Secretor status, candidal carriage and candidal infection in patients with diabetes mellitus. J Oral Pathol.

[15] Kadir T, Pisiriciler R, Akyüz S, Yarat A, Emekli N, Ipbüker A (2002). Mycological and cytological examination of oral candidal carriage in diabetic patients and non-diabetic control subjects: Thorough analysis of local aetiologic and systemic factors. J Oral Rehabil.

[16] Al-Karaawi ZM, Manfredi M, Waugh AC, McCullough MJ, Jorge J, Scully C (2002). Molecular characterization of *Candida spp*. isolated from the oral cavities of patients from diverse clinical settings. Oral Microbiol Immunol.

[17] Belazi M, Velegraki A, Fleva A, Gidarakou I, Papanaum L, Baka D (2005). Candidal overgrowth in diabetic patients: Potential predisposing factors. Mycoses.

[18] Manfredi M, McCullough MJ, Al-Karaawi ZM, Vescovi P, Porter SR (2006). Analysis of the strain relatedness of oral *Candida albicans* in patients with diabetes mellitus using polymerase chain reaction-fingerprinting. Oral Microbiol Immunol.

[19] Fisher BM, Lamey PJ, Samaranayake LP, MacFarlane TW, Frier BM (1987). Carriage of *Candida* species in the oral cavity in diabetic patients: Relationship to glycaemic control. J Oral Pathol.

[20] Darwazeh AM, Lamey PJ, Samaranayake LP, MacFarlane TW, Fisher BM, Macrury SM (1990). The relationship between colonisation, secretor status and in-vitro adhesion of *Candida albicans* to buccal epithelial cells from diabetics. J Med Microbiol.

[21] Borromeo GL, McCullough MJ, Reade PC (1992). Quantitation and morphotyping of *Candida albicans* from healthy mouthsand from mouths affected by erythematous candidosis. J Med Vet Myco.

[22] Sahin I, Oksuz S, Sencan I, Gulcan A, Karabay O, Gulcan E (2005). Prevalance and risk factors for yeast colonization in adult diabetic patients. Ethiop Med J.

[23] Odds F (1988). *Candida* and candidosis. A review and bibliography.

[24] Fongsmut T, Deerochanawong C, Prachyabrued W (1998). Intraoral *Candida* in Thai diabetes patients. J Med Assoc Thai.

[25] Nowakowska D, Kurnatowska A, Stray-Pedersen B, Wilczyñski J (2004). Species distribution and influence of glycemic control on fungal infections in pregnant women with diabetes. J Infect.

[26] Gonçalves RH, Miranda ET, Zaia JE, Giannini MJ (2006). Species diversity of yeast in oral colonization of insulin-treated diabetes mellitus patients. Mycopathologia.

[27] Manfredi M, McCullough MJ, Polonelli L, Conti S, Al-Karaawi ZM, Vescovi P (2006). In vitro antifungal susceptibility to six antifungal agents of 229 *Candida* isolates from patients with diabetes mellitus. Oral Microbiol Immunol.

[28] Diaz-Guerra TM, Martinez-Suarez JV, Laguna F, Valencia E, Rodriguez-Tudela JL (1998). Change in fluconazole susceptibility patterns and genetic relationship among oral *Candida albicans isolates*. AIDS.

[29] Rex JH, Pfaller MA, Walsh TJ, Chaturvedi V, Espinel-Ingroff A, Ghannoum MA (2001). Antifungal susceptibility testing: Practical aspects and current challenges. Clin Microbiol Rev.

[30] Morace G, Amato G, Bistoni F, Fadda G, Marone P, Montagna MT (2002). Multicenter comparative evaluation of six commercial systems and the national committee for clinical laboratory standards m27-a broth microdilution method for fluconazole susceptibility testing of *Candida* species. J Clin Microbiol.

[31] Guggenheimer J, Moore PA, Rossie K, Myers D, Mongelluzzo MB, Block HM (2000). Insulin dependent diabetes mellitus and oral soft tissue pathologies: II, Prevalence and characteristics of Candida and Candidal lesions. Oral Surg Oral Med Oral Pathol Oral Radiol Endod.

